# Development of examination objectives for the Korean paramedic and emergency medical technician examination: a survey study

**DOI:** 10.3352/jeehp.2024.21.13

**Published:** 2024-06-12

**Authors:** Tai-hwan Uhm, Heakyung Choi, Seok Hwan Hong, Hyungsub Kim, Minju Kang, Keunyoung Kim, Hyejin Seo, Eunyoung Ki, Hyeryeong Lee, Heejeong Ahn, Uk-jin Choi, Sang Woong Park

**Affiliations:** 1 Department of Paramedicine, Eulji University, Seongnam, Korea; 2 Department of Paramedicine, Kyungil University, Gyongsan, Korea; 3 Department of Paramedicine, Jeonju Kijeon College, Jeonju, Korea; 4 Department of Paramedicine, Daegu Health College, Daegu, Korea; 5 Department of Paramedicine, Ansan University, Ansan, Korea; 6 Department of Physiology, Institute of Functional Genomics, & Research Institute of Medical Science, Konkuk University School of Medicine, Chungju, Korea; 7 Department of Paramedicine, Yeonsung University, Anyang, Korea; 8 Department of Paramedicine, Seojeong University, Yangju, Korea; Hallym University, Korea

**Keywords:** Ambulances, Cardiopulmonary resuscitation, Emergency medical technicians, Paramedics, Republic of Korea

## Abstract

**Purpose:**

The duties of paramedics and emergency medical technicians (P&EMTs) are continuously changing due to developments in medical systems. This study presents evaluation goals for P&EMTs by analyzing their work, especially the tasks that new P&EMTs (with less than 3 years’ experience) find difficult, to foster the training of P&EMTs who could adapt to emergency situations after graduation.

**Methods:**

A questionnaire was created based on prior job analyses of P&EMTs. The survey questions were reviewed through focus group interviews, from which 253 task elements were derived. A survey was conducted from July 10, 2023 to October 13, 2023 on the frequency, importance, and difficulty of the 6 occupations in which P&EMTs were employed.

**Results:**

The P&EMTs’ most common tasks involved obtaining patients’ medical histories and measuring vital signs, whereas the most important task was cardiopulmonary resuscitation (CPR). The task elements that the P&EMTs found most difficult were newborn delivery and infant CPR. New paramedics reported that treating patients with fractures, poisoning, and childhood fever was difficult, while new EMTs reported that they had difficulty keeping diaries, managing ambulances, and controlling infection.

**Conclusion:**

Communication was the most important item for P&EMTs, whereas CPR was the most important skill. It is important for P&EMTs to have knowledge of all tasks; however, they also need to master frequently performed tasks and those that pose difficulties in the field. By deriving goals for evaluating P&EMTs, changes could be made to their education, thereby making it possible to train more capable P&EMTs.

## Graphical abstract

[Fig f3-jeehp-21-13]

## Introduction

### Background/rationale

According to the Korean Emergency Medical Services Act, paramedics and emergency medical technicians (P&EMTs) can engage in first aid work at the scene of an incident, during transportation, and within medical institutions. P&EMTs are key personnel who provide high-level first aid and counseling to patients on-site and deliver patient information to hospital-based medical personnel.

The competencies of P&EMTs include the ability to provide consultation, rescue, transport, and first aid to emergency patients, as well as invasive first aid through direct medical guidance, patient evaluation, and management of the emergency medical service system [1]. P&EMTs have recently entered diverse fields, such as private organizations and the Coast Guard, and must be able to treat patients in various environments [2]. P&EMTs must possess a variety of abilities; therefore, it is necessary to train P&EMTs who are experts in performing complex tasks related to saving patients’ lives. To qualify as P&EMTs, candidates must pass an examination administered by the Korea Health Personnel Licensing Examination Institute (KJPLEI), which oversees emergency medical technician qualification tests. The KJPLEI must establish a framework for P&EMTs’ qualification examinations. In particular, because P&EMTs work in a variety of jobs, various patients and work environments must be evaluated.

Therefore, this study investigated the tasks that P&EMTs currently perform, those that they consider important, and those that they consider difficult through a comprehensive job analysis. The current curriculum of P&EMT Departments is taught through a national exam. Therefore, deriving goals for evaluating P&EMTs would be helpful in training P&EMTs to respond to a variety of medical emergencies.

### Objectives

This study aimed to present the evaluation goals necessary for the national examination by analyzing the work of P&EMTs. In addition, we present tasks that new P&EMTs find difficult to perform. Therefore, analyzing the characteristics of the duties of P&EMTs will help educate and evaluate P&EMTs who could respond to any medical emergency.

## Methods

### Ethics statement

This study was approved by the Institutional Review Board of Eulji University (EUIRB2023-058). Informed consent was obtained from all participants. This study conformed to the guidelines for the study of humans and animals stipulated in the Declaration of Helsinki.

### Study design

This study was qualitative in nature. A survey was conducted among qualified P&EMT members from July to October 2023. The survey investigated the frequency, importance, and difficulty of 9 duties, 54 tasks, and 253 task elements implemented in the field by 448 participants. Dataset 1 presents the questionnaire results.

### Setting

**Fig. 1.**
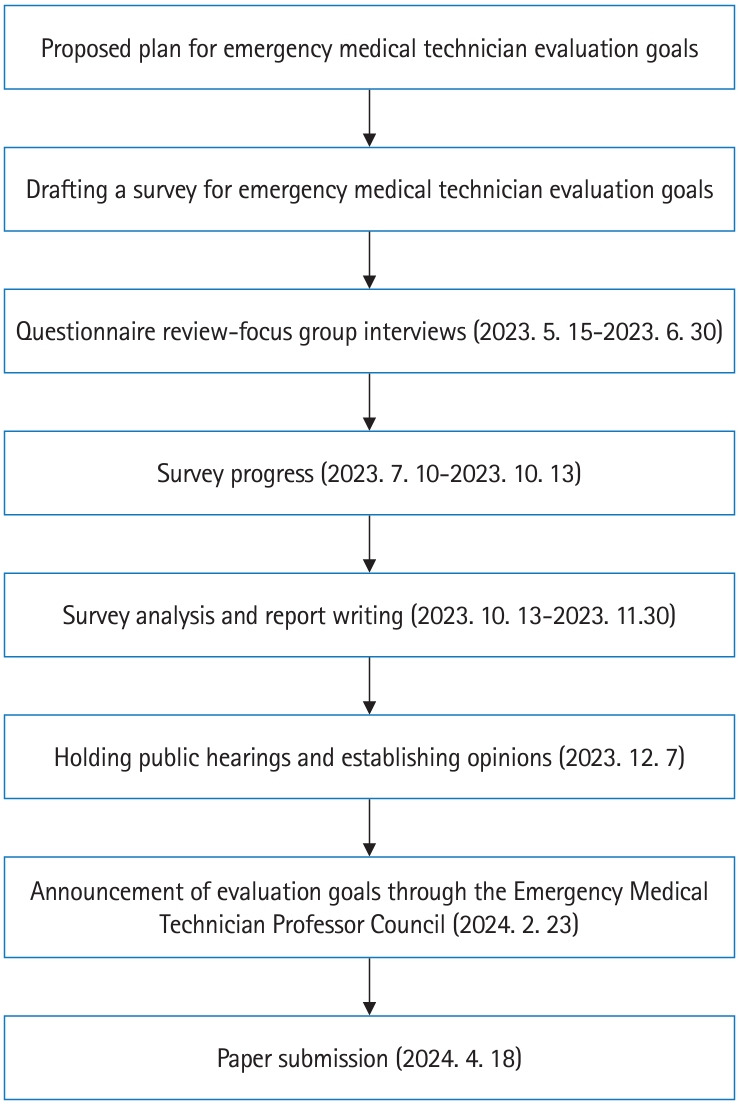
Flow of the study.

This study was based on 2 prior job analyses to create evaluation goals for the P&EMT. The previous study was limited to 2 occupations; however, here, data were collected from participants engaged in 6 occupations in which P&EMTs currently work. In addition, the tasks currently performed by P&EMTs were incorporated into this research. The questionnaire was modified through focus group interviews. Survey analyses were conducted through public hearings to establish opinions, and guidance was provided by the faculty council. Fig. 1 shows the study’s overall flowchart.

### Participants

This study’s participants were P&EMTs. A survey was administered to P&EMTs from 6 areas where P&EMTs are currently employed: fire departments, hospitals, industry, coast guard, military, and others (mountain rescue teams, civil servants, etc.). For paramedics, the researcher explained the survey contents and received the survey through a paper questionnaire, and 199 people completed the survey. For the EMTs, contact information was received through the EMTs’ Association, and a Google Form was sent via the internet to 1,200 people. Among them, 249 completed the survey. Table 1 lists the age and sex of the participants. A total of 448 (199+249) participants responded to the survey.

### Variables

The main results were the scale used in the questionnaire.

### Data sources/measurement

Before 2023, 2 surveys were administered to EMTs. The first was conducted in 2000 (166 questions) and the second in 2013 (240 questions). For the second survey, questionnaire questions were created using the developing a curriculum process analysis technique [3]. The current research was based on a third survey. In this survey, questions were created by revising and securing the contents of the first and second surveys and were verified after recruiting a group of experts (253 questions) (Table 2).

This study solicited answers to 253 questions for 6 job positions. Paramedics primarily work in fire departments or hospitals, whereas EMTs work in fire departments or the military. The overall work frequency, importance, and difficulty levels were assessed for all paramedics, and the comparison between new and experienced paramedics was divided into P&EMTs. The goal of the study was to survey 480 people: 40 from each occupational group (240 paramedics and 240 EMTs). However, most of the participants worked in fire departments and hospitals, and only paramedics (no EMTs) worked as marine rescuers. In addition, the military employs more EMTs than paramedics. This study’s goal was to identify the abilities needed in the current job field, and Supplement 1 provides the survey information for each job group. This survey used a 6-point Likert-type scale, with 1 point=0%–10%, 2 points=11%–30%, 3 points=31%–50%, 4 points=51%–70%, and 5 points=71%–90%. A score of 6 points was recorded as 91%–100%.

### Bias

This study presents participants’ individual value judgments for each job, which might vary depending on the job. In addition, second-level paramedics mainly perform duties other than actual emergency services because they have obtained qualifications from the fire academy and military.

### Study size

The study size was not estimated since this was a descriptive study. The authors conventionally selected the targets.

### Statistical methods

The data were analyzed using PASW SPSS ver. 18.0 (SPSS Inc.). The results were obtained via Pearson correlation analysis of the average values of frequency, importance, and difficulty, without distinguishing between P&EMTs. In addition, to compare new and experienced paramedics versus EMTs, the independent sample t-test was conducted.

## Results

### Participants

Table 1 presents the characteristics of the participants, and Fig. 1 shows the stages of the study. A survey was conducted with 448 people. We collected the opinions of 8 external experts regarding this study’s results through public hearings.

### Main results

#### Analysis of P&EMTs’ curriculum and question scope in the national examination for P&EMTs

To analyze the learning goals for each of the subjects taken by the P&EMTs, the final subject names were derived by analyzing the scope of the questions for the 5 subjects in the national examination (Table 3) and the connections between those subjects derived from 15 schools. Those who have received a regular degree from a university or college could take the paramedics exam, and EMTs can take the examination after completing at least 243 hours of theory and 100 hours of practical training at an institution. The subjects studied by the paramedic students were related to the national examination, while those unrelated to the paramedics’ national examination included water lifesaving, research methodology, and fire science. Students training to become EMTs studied topics related to the national examination only.

#### Questionnaire-based study (P&EMTs’ job frequency-importance-difficulty)

In the survey involving 448 P&EMTs, the mean±standard deviation for the 253 questions were as follows: frequency 3.52±2.062, importance 5.10±1.321, and difficulty 3.60±1.565 on paramedics, and frequency 3.11±1.776, importance 4.05±1.647, and difficulty 3.41±1.584 on EMTs. We calculated which task elements the P&EMTs performed the most, which tasks they considered important, and which tasks they found difficult to perform. The paramedics reported that the task they were most frequently responsible for was taking chief complaints, whereas the most important task for paramedics was related to advanced cardiovascular life support for cardiac arrest. The most difficult areas were found to be difficulties in taking a pertinent history. The EMTs reported that the task they were most frequently responsible for was checking body temperature, whereas the most important task for paramedics was applying an automated external defibrillator (AED). The most difficult area was difficulties in treating neonatal cardiac arrest (Tables 4–6). Dataset 1 presents all the survey data.

#### Frequency-importance-difficulty correlation analysis based on the job analysis

**Fig. 2.**
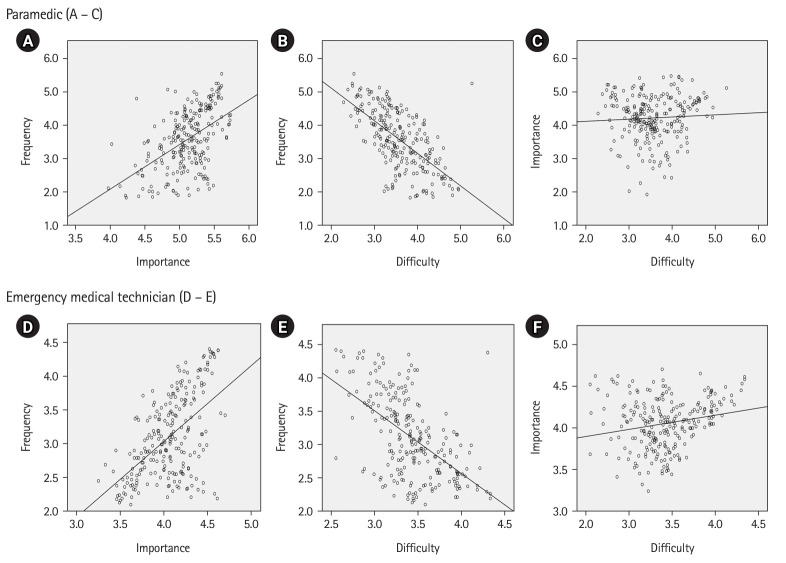
Analysis of emergency medical technician work. Data on 253 work factors were analyzed using Pearson correlation analysis. (A–C) present the analysis for paramedics: (A) Frequency-importance correlation analysis through job analysis. The y-axis represents frequency, and the x-axis represents importance. It is significant at P<0.001, and the Pearson correlation coefficient is 0.504. (B) Frequency difficulty correlation analysis through job analysis. The y-axis represents frequency, and the x-axis represents difficulty. It is significant at P<0.001, and the Pearson correlation coefficient is 0.638. (C) Importance and difficulty based on a job analysis. The y-axis represents importance, and the x-axis represents difficulty. It is significant at P<0.330, and the Pearson correlation coefficient is 0.064, indicating no correlation. (D–F) were analysis on emergency medical technicians: (D) Frequency-importance correlation analysis through job analysis. The y-axis represents frequency, and the x-axis represents importance. It is significant at P<0.001, and the Pearson correlation coefficient is 0.556. (E) Frequency-difficulty correlation analysis through job analysis. The y-axis represents frequency, and the x-axis represents difficulty. It is significant at P<0.001, and the Pearson correlation coefficient is -0.564. (F) Importance and difficulty based on a job analysis. The y-axis represents importance, and the x-axis represents difficulty. It is significant at P<0.001, and the Pearson’s correlation coefficient is 0.0207, indicating no correlation.

A statistically significant positive correlation was found between importance and frequency, with Pearson correlation coefficients of 0.504 for paramedics and 0.556 for EMTs (Fig. 2A, D and Dataset 2). A statistically significant negative correlation was found between difficulty and frequency, with a Pearson correlation coefficient of -0.638 for paramedics and -0.564 for EMTs (Fig. 2A, D). There was no correlation between difficulty and importance, with Pearson correlation coefficients of 0.064 for paramedics and 0.207 for EMTs, and the importance of the task elements was found to be unrelated to the difficulty level (Fig. 2C, F).

#### Difficulty analysis among new and experienced P&EMTs

All P&EMTs responded that emergency deliveries and pediatric cardiopulmonary resuscitation (CPR) were difficult tasks. The new paramedics responded that drug addiction, childhood fever, and bone marrow were difficult (Table 7), whereas the new EMTs responded that emergency logs, ambulance management, and infection control were difficult (Table 8).

## Discussion

### Key results

This study’s goal was to investigate the work performed by P&EMTs and to reflect the results of national examinations to facilitate smooth work. An additional goal of this study was to provide guidelines for training P&EMT who could be deployed in the field immediately after obtaining a certification. The task elements with high importance included communication with patients, such as applying an AED, conducting CPR, providing relief from choking, and taking the chief complaint. CPR is a skill related to life preservation and is considered important, as it is an item with which P&EMTs must be fully familiar.

### Interpretation

It has been reported that CPR training increases CPR knowledge, and that considerable experience is important. In addition, it has been reported that upper limb muscle mass and body weight are important for CPR. Therefore, P&EMTs consider continuous CPR training and physical strength to be important elements of their work.

High-frequency tasks were evaluated as relatively easy, whereas low-frequency tasks were rated as difficult. The difficult tasks were general and emergency deliveries, as well as the treatment of neonatal or child cardiac arrest. In addition, P&EMTs with less than 3 years of work experience were compared with P&EMTs with more than 3 years of work experience. Paramedics were found to have great difficulty in treating injection poisoning, drug overdoses, fractures and dislocations, sprains and strains, and children with fever. It is believed that EMTs who have more experience with patients could perform their work more smoothly. If school studies focused on these tasks, new EMTs would be able to resolve the difficulties of their work. EMTs were found to have difficulty documenting pre-hospital care reports, disinfecting ambulances, and responding to infectious diseases. It was found that the training time for EMTs was short: 2 hours for documenting pre-hospital care reports, 5 hours for responding to infectious diseases, and 0 hours for disinfecting ambulances. They responded that common difficulties faced by P&EMTs involved providing treatment related to the heart, brain, or delivery. Paramedics needed in-depth learning and EMTs needed to add the ability to assist paramedics.

This study examined the task elements of P&EMTs. In Korea, P&EMTs, who were previously only responsible for transporting patients, have now become active in various fields; therefore, their scope of work had to be expanded. The core goal of the national P&EMT examination is to develop P&EMTs who could perform fieldwork in response to changing emergency medical environments. Therefore, an evaluation goal was necessary.

### Comparison with previous studies

The previous national examination for P&EMTs was based on P&EMTs working only in hospitals and fire departments [3]. However, owing to the expansion of P&EMTs’ duties, surveys were needed in various field contexts; therefore, we conducted a survey targeting P&EMTs working in the military, private industry, and the Coast Guard. In addition, a previous study conducted a survey targeting 2 fire departments, suggesting limitations in generalizability. However, in this study, the survey targeted fire stations nationwide.

The duties of P&EMTs include the delivery of newborns. We examined the reasons behind the difficulties encountered during this task. Education on newborn delivery often emphasizes theoretical learning. This issue is not unique to Korea; other countries have also reported a lack of delivery-related education for paramedics [4].

### Limitations

This study conducted a survey of various occupations, focusing primarily on paramedics and EMTs. Most paramedics work in fire departments and hospitals, while the majority of EMTs are employed in fire departments. This trend is likely because many EMT training programs are conducted at fire academies. The research results in the frequency section may not represent all occupations comprehensively. Although this study cannot present results for all occupations, it aims to improve the education of paramedics and EMTs based on difficulty analyses. It is believed that this improvement is achievable.

### Generalizability

The purpose of this study was to propose directions for the development of P&EMTs’ education and to guide the direction of national examinations. P&EMTs worldwide perform work directly related to the lives of patients in the field, necessitating a wide range of knowledge and skills. The types of patient incidents may vary by country, making it essential to train high-quality P&EMTs by examining the frequency of different patient circumstances. Recent research has shown that the older population (65 years and above) of many other countries is gradually increasing. It is important to establish a medical system suited to the aging population [5]. P&EMTs tend to have difficulty communicating with older patients, which has been attributed to a lack of older adult-related education and practical training [6]. Furthermore, smooth communication with not only patients, but also other medical personnel is a competency that P&EMTs must possess. This might apply not only to Korea, but also to other countries [7].

### Suggestions

In Korea, P&EMTs mostly work in hospitals and fire departments. In addition, P&EMTs must collaborate with various professionals with health-related licenses, not just with other P&EMTs. Future studies should examine the necessity of collaboration between occupations. In particular, a plan for work collaboration among P&EMTs is required.

### Conclusion

It was not possible to test P&EMTs for all tasks that they performed. However, high-quality P&EMTs could be trained through tests on important and frequent tasks in paramedical work. Furthermore, more work experience regarding a specific task was associated with a lower level of reported difficulty. This implies that experience is important. A significant amount of experience could be acquired in schools. The development of examination objectives could lead to changes in school education, and P&EMTs with professional knowledge and skills could be trained.

## Figures and Tables

**Figure f3-jeehp-21-13:**
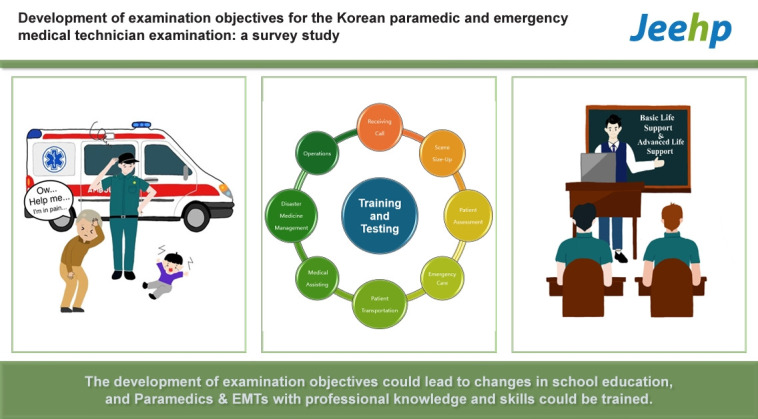


**Table 1.** Participants’ demographic characteristics

Characteristic	Paramedics	EMTs	Overall
Respondents	199 (44.7)	249 (55.3)	448 (100.0)
Age (yr)	31.73±6.769	37.88±6.332	35.13±7.200
Post-acquisition related experience (yr)	7.41±5.970	4.61±3.753	5.87±5.061
Gender			
Male	109 (54.8)	234 (94.3)	343
Under 3 years^a)^	23 (33.8)	45 (66.2)	68
Over 3 years^b)^	86 (31.5)	188 (68.5)	274
No answer		1	1
Female	90 (45.2)	15 (5.7)	105 (23.4)
Under 3 years	34 (91.9)	3 (8.1)	37
Over 3 years	56 (83.6)	12 (16.4)	68

Values are presented as number (%) or mean±standard deviation. EMTs, emergency medical technicians.

a) Under 3 years: within 3 years of obtaining paramedic or EMT certification.

b) Over 3 years: after 3 years of obtaining paramedic or EMT certification.

**Table 2.** Job analysis for paramedics and emergency medical technicians

Year	Duty	Task	Task element	Note
First study (2000)	9	48	166	- The first job analysis (112 respondents)
				- Workshop (12 researchers)
Second study (2013)	10	57	240	- The second job analysis (330 respondents)
				- Adding duties related to disaster medicine management
				- Supplementing duty physician assistant (4 tasks & 45 task elements)
				- DACUM based on the first job analysis (8 researchers & 10 FGIs)
This study (2023)	9	54	253	- The third job analysis (445 respondents)
				- Putting duties related to going to the scene into operation
				- Supplementing duties related to disaster medicine management (6 tasks & 28 task elements)
				- Literature review based on the first and second job analyses (11 researchers & 9 FGIs)

DACUM, developing a curriculum process; FGIs, focus group interviews.

**Table 3.** Subjects for paramedics and emergency medical technicians (National Registry Cognitive Exam)

Subjects for paramedics	Domains
Basic medicine	Cells and tissues, systems, infections
Emergency patient management	Emergency patient evaluation, emergency patient management and medical assisting
Professional EMS operations	Introduction to EMS, rescue and transport, disaster management
Professional emergency care	Cardiology, trauma, medical, special emergency
Laws	Emergency Medical Service Act, Medical Service Act
Subjects for EMTs	Domains
Emergency patient management	Emergency patient evaluation, emergency patient management and medical assisting
Basic EMS operations	Introduction to EMS, rescue and transport, disaster management
Basic emergency care	Cardiology, trauma, medical, special emergencies
Laws	Emergency Medical Service Act, Medical Service Act

EMS, emergency medical service.

**Table 4.** Top 10 task elements in terms of frequency

	Mean±SD
Task elements for paramedics	
Identifying nature of call	4.58±1.922
Identifying location of patient	4.46±1.936
Identifying number of patients	4.29±1.920
Identifying hazmat	3.72±1.857
Identifying required resources	4.17±1.869
Identifying patient’s chief complaint	4.88±1.754
Identifying patient’s age and sex	4.7±1.807
Determining priority of response	4.16±4.895
Informing about the patient’s state	4.03±2.031
Informing about the condition at the scene	4.05±2.021
Task elements for EMTs	
Checking body temperature	4.40±1.691
Checking SPO_2_	4.38±1.724
Taking chief complaint	4.38±1.767
Taking pertinent history	4.36±1.737
Checking pulse	4.35±1.764
Identifying patient’s chief complaint	4.33±1.720
Securing patient’s safety	4.33±1.614
Taking past history	4.30±1.750
Obtaining information about the scene	4.28±1.630
Checking BP	4.28±1.755

SD, standard deviation; EMS, emergency medical service; SPO_2_, saturation of percutaneous O_2_; BP, blood pressure.

**Table 5.** Top 10 task elements in terms of importance

	Mean±SD
Task elements for paramedics	
ACLS for cardiac arrest	5.74±0.708
ACLS for imminent cardiac arrest	5.73±0.738
CPR and relief of choking	5.72±0.769
ACLS for MI	5.72±0.756
Applying AED	5.71±0.768
ACLS for non-cardiogenic cardiac arrest	5.68±0.786
ACLS for ROSC	5.68±0.826
Treating stroke	5.62±0.887
Assessing and maintaining airway	5.61±0.745
Checking pulse	5.61±0.754
Task elements for EMTs	
Applying AED	4.70±1.566
CPR and relief of chocking	4.65±1.571
Checking SPO_2_	4.62±1.566
Taking chief complaint	4.62±1.569
Treating neonatal cardiac arrest	4.61±1.607
Checking BP	4.58±1.568
Treating child cardiac arrest	4.58±1.650
Securing patient’s safety	4.57±1.510
Identifying patient’s chief complaint	4.56±1.521
Checking pulse	4.55±1.616

SD, standard deviation; ACLS, advanced cardiovascular life support; CPR, cardiopulmonary resuscitation; MI, myocardial infarction; AED, automated external defibrillator; ROSC, return of spontaneous circulation; EMS, emergency medical service; EMT, emergency medical technician; SPO_2_, saturation of percutaneous O_2_; BP, blood pressure.

**Table 6.** Top 10 task elements in terms of difficulty

	Mean±SD
Task elements for paramedics	
Taking pertinent history	5.26±1.314
Managing emergency labor and delivery	4.95±1.221
Managing labor and delivery	4.94±2.523
Treating neonatal cardiac arrest	4.81±1.381
Treating eclampsia	4.81±1.278
Treating cardiac tamponade	4.77±1.226
Treating child cardiac arrest	4.74±1.397
Treating open chest injury	4.64±1.256
Treating hemothorax	4.64±1.267
Treating abdominal injury	4.62±1.356
Task elements for EMTs	
Treating neonatal cardiac arrest	4.33±1.655
Treating child cardiac arrest	4.33±1.650
Taking pertinent history	4.3±1.750
Managing emergency labor and delivery	4.29±1.633
Treating brain injury	4.21±1.870
Treating abdominal injury	4.18±1.655
Treating open chest injury	4.18±1.627
ACLS for ROSC	4.11±1.617
ACLS for non-cardiogenic cardiac arrest	4.09±1.661
Treating pulmonary contusion	4.07±1.681

SD, standard deviation; EMT, emergency medical technician; ACLS, advanced cardiovascular life support; ROSC, return of spontaneous circulation.

**Table 7.** Difficult task elements for new paramedics

	Independent-samples test for paramedics							
	Levene’s test for equality of variances		t-test for equality of mean					
	F-value	Sig.	t-value	df	Sig.	MD	SED	95% CI of the difference
Treating injection poisoning								
Assumed	4.493	0.035	-2.003	186	0.047	-0.43256	0.21598	-0.85865 to -0.00647
Not assumed			-2.274	131.885	0.025	-0.43256	0.19018	-0.80876 to -0.05636
Treating drug overdose								
Assumed	0.004	0.953	-2.914	185	0.004	-0.57004	0.19565	-0.95602 to -0.18405
Not assumed			-3.043	108.199	0.003	-0.57004	0.18734	-0.94137 to -0.19870
Treating fracture and dislocation								
Assumed	0.001	0.979	-1.972	187	0.050	-0.46228	0.23437	-0.92462 to 0.00006
Not assumed			-1.922	95.184	0.058	-0.46228	0.24054	-0.93980 to 0.01524
Treating sprain and strain								
Assumed	0.065	0.800	-3.009	187	0.003	-0.74043	0.24608	-1.22589 to -0.25498
Not assumed			-2.980	98.479	0.004	-0.74043	0.24848	-1.23351 to -0.24736
Treating childhood fever								
Assumed	0.906	0.342	-2.428	187	0.016	-0.60000	0.24710	-1.08746 to -0.11254
Not assumed			-2.507	104.669	0.014	-0.60000	0.23935	-1.07460 to -0.12540

Sig., significance; df, degrees of freedom; MD, mean difference; SED, standard error difference; CI, confidence interval.

**Table 8.** Difficult task elements for new emergency medical technicians

	Independent samples test for emergency medical technicians							
	Levene’s test for equality of variances		t-test for equality of mean					
	F-value	Sig.	t-value	df	Sig.	MD	SED	95% CI of the difference
Documenting pre-hospital care report								
Assumed	4.493	0.035	-2.003	186	0.047	-0.43256	0.21598	-0.85865 to -0.00647
Not assumed			-2.274	131.885	0.025	-0.43256	0.19018	-0.80876 to -0.05636
Disinfecting ambulance								
Assumed	0.004	0.953	-2.914	185	0.004	-0.57004	0.19565	-0.95602 to -0.18405
Not assumed			-3.043	108.199	0.003	-0.57004	0.18734	-0.94137 to -0.19870
Responding to infectious disease								
Assumed	0.001	0.979	-1.972	187	0.050	-0.46228	0.23437	-0.92462 to 0.00006
Not assumed			-1.922	95.184	0.058	-0.46228	0.24054	-0.93980 to 0.01524

Sig., significance; df, degrees of freedom; MD, mean difference; SED, standard error difference; CI, confidence interval.

## Footnotes

**Data availability**
Data files are available from Harvard Dataverse: https://doi.org/10.7910/DVN/BSUYWS Dataset 1. Raw data of participants’ responses to the questionnaires.

Dataset 2. Tasks of paramedics and emergency medical technicians and their frequency, importance, and difficulty.

## Supplementary materials

Supplementary files are available from Harvard Dataverse: https://doi.org/10.7910/DVN/BSUYWS

Supplement 1. Survey questionnaires for development of examination objectives for the Korean paramedic and emergency medical technician examination.

Supplement 2. Audio recording of the abstract.
