# Supplementary material for: Development of examination objectives for the Korean paramedic and emergency medical technician examination: a survey study
Source: J Educ Eval Health Prof. 2024 Jun 12;21:13. doi: 10.3352/jeehp.2024.21.13 (PMC11239538; doi:10.3352/jeehp.2024.21.13)
Supplement: Supplementary file 3 — Supplement 1. Survey questionnaires for development of examination objectives for the Korean paramedic and emergency medical technician examination. [file jeehp-21-13-suppl1.pdf]

This is a survey Receiving Call. Please mark “○” for the questions that apply according to the criteria below.

Frequency					
①	②	③	④	⑤	⑥
0-10 %	11-30 %	31-50 %	51-70 %	71-90 %	90-100 %

Importance					
①	②	③	④	⑤	⑥
0-10 %	11-30 %	31-50 %	51-70 %	71-90 %	90-100 %

Difficulty					
①	②	③	④	⑤	⑥
0-10 %	11-30 %	31-50 %	51-70 %	71-90 %	90-100 %

Duty Ⅰ																			
Receiving Call																			
Task	Task Elements	Frequency						Importance						Difficulty					
		①	②	③	④	⑤	⑥	①	②	③	④	⑤	⑥	①	②	③	④	⑤	⑥
1.1. Identifying scene	Identifying nature of call																		
	Identifying location of patient																		
	Identifying number of patient																		
	Identifying hazmat																		
	Identifying required resources																		
1.2. Identifying patient	Identifying patient’s chief complaint																		
	Identifying patient’s age & sex																		
1.3. Guiding response	Determining priority of response																		
	Informing state of patient																		
	Informing condition of scene																		
	Requesting additional resources																		
1.4. Pre-arrival instructions	Informing basic CPR																		
	Informing AED																		
	Informing first aid																		
	Informing scene safety																		

This is a survey for Scene Size-Up. Please mark "○" for the questions that apply according to the criteria below.

Frequency					
①	②	③	④	⑤	⑥
0-10 %	11-30 %	31-50 %	51-70 %	71-90 %	90-100 %

Importance					
①	②	③	④	⑤	⑥
0-10 %	11-30 %	31-50 %	51-70 %	71-90 %	90-100 %

Difficulty					
①	②	③	④	⑤	⑥
0-10 %	11-30 %	31-50 %	51-70 %	71-90 %	90-100 %

Duty Ⅱ																			
Scene Size-Up																			
Task	Task Elements	Frequency						Importance						Difficulty					
		①	②	③	④	⑤	⑥	①	②	③	④	⑤	⑥	①	②	③	④	⑤	⑥
2.1. Communicating on enroute	Obtaining information of scene																		
	Informing condition of traffic																		
	Informing ETA																		
2.2. Securing scene safety	Securing personal safety																		
	Securing patient's safety																		
	Securing bystander's & co-worker's safety																		
	Requesting additional help																		

This is a survey for Patient Assessment. Please mark "○" for the questions that apply according to the criteria below.

Frequency					
①	②	③	④	⑤	⑥
0-10 %	11-30 %	31-50 %	51-70 %	71-90 %	90-100 %

Importance					
①	②	③	④	⑤	⑥
0-10 %	11-30 %	31-50 %	51-70 %	71-90 %	90-100 %

Difficulty					
①	②	③	④	⑤	⑥
0-10 %	11-30 %	31-50 %	51-70 %	71-90 %	90-100 %

Duty Ⅲ																			
Patient Assessment																			
Task	Task Elements	Frequency						Importance						Difficulty					
		①	②	③	④	⑤	⑥	①	②	③	④	⑤	⑥	①	②	③	④	⑤	⑥
3.1. Initial assessment	Forming general impression & mental status																		
	Assessing & immobilizing neck																		
	Assessing & maintaining airway																		
	Assessing breathing & delivering O2																		
	Assessing circulation & delivering fluid																		
	Assessing disability																		
	Assessing mechanism of injury																		
	Determining priority of patient																		
3.2. Secondary assessment	Assessing head & face																		
	Assessing neck																		
	Assessing thorax																		
	Assessing abdomen																		
	Assessing pelvis																		
	Assessing extremities																		
	Assessing back																		
3.3. Checking vital sign	Checking BP																		
	Checking pulse																		

	Checking respiration																		
	Checking body temperature																		
	Checking SPO2																		
	Checking ECG																		
3.4. History taking	Taking chief complaint																		
	Taking pertinent history																		
	Taking past history																		
	Taking family history																		
	Taking social history																		

This is a survey for Emergency Care. Please mark “○” for the questions that apply according to the criteria below.

Frequency					
①	②	③	④	⑤	⑥
0-10 %	11-30 %	31-50 %	51-70 %	71-90 %	90-100 %

Importance					
①	②	③	④	⑤	⑥
0-10 %	11-30 %	31-50 %	51-70 %	71-90 %	90-100 %

Difficulty					
①	②	③	④	⑤	⑥
0-10 %	11-30 %	31-50 %	51-70 %	71-90 %	90-100 %

Duty Ⅳ																			
Emergency Care																			
Task	Task Elements	Frequency						Importance						Difficulty					
		①	②	③	④	⑤	⑥	①	②	③	④	⑤	⑥	①	②	③	④	⑤	⑥
4.1. Basic CPR	CPR & relief of chocking																		
	Applying AED																		
4.2. ACLS	ACLS on cardiac arrest																		
	ACLS on imminent cardiac arrest																		
	ACLS on MI																		
	ACLS on non-cardiogenic cardiac arrest																		
	ACLS on ROSC																		
4.3. Respiratory system	Treating COPD																		
	Treating asthma																		
	Treating hyperpnea																		
	Treating hemoptysis																		
4.4. Cardiovascular system	Treating chest pain																		
	Treating dysrhythmia																		
4.5. Endocrine system	Treating metabolic disorder																		
	Treating diabetic emergency																		
4.6. Nervous system	Treating stroke																		
	Treating fainting seizure epilepsy																		

4.7. Digestive urinary reproductive system	Treating gastrointestinal patients																		
	Treating digestive emergency																		
	Treating urologic emergency																		
	Treating reproductive emergency																		
4.8. Anaphylaxis	Treating allergy																		
	Treating anaphylaxis																		
4.9. Contagion	Treating septic shock																		
	Treating infection																		
4.10. Poisoning	Treating inhalation poisoning																		
	Treating absorption poisoning																		
	Treating injection poisoning																		
	Treating ingested poisoning																		
	Treating drug overdose																		
	Treating alcoholism																		
4.11. Bleeding	Treating hemostasis																		
	Treating hemorrhagic shock																		
4.12. Injury on head, neck & face	Treating scalp injury																		
	Treating skull fracture																		
	Treating brain injury																		
	Treating eye injury																		
	Treating teeth injury																		
4.13. Injury on thorax	Treating pneumothorax																		
	Treating hemothorax																		
	Treating frail chest																		
	Treating cardiac tamponade																		
	Treating rib fracture																		

	Treating open chest injury																		
	Treating pulmonary contusion																		
4.14.Injury on spine	Treating cervical spine injury																		
	Treating spine injury																		
	Treating neurologic shock																		
4.15. Injury on abdomen	Treating blunt trauma																		
	Treating penetration & puncture																		
	Treating abdominal injury																		
4.16. Injury on pelvis	Treating pelvic fracture																		
	Treating urologic & genital injury																		
4.17. Injury on bone	Treating fracture & dislocation																		
	Treating sprain & strain																		
4.18.Injury on soft tissue	Treating bleeding																		
	Treating wound																		
	Treating open wound																		
	Treating closed wound																		
4.19. Burn	Treating thermal burn																		
	Treating inhalation burn																		
	Treating chemical burn																		
	Treating electricity burn																		
4.20. Ob & Gy emergency	Managing emergency labor & delivery																		
	Managing labor & delivery																		
	Treating eclampsia																		
4.21. Infant & child emergency	Treating neonatal cardiac arrest																		
	Treating child cardiac arrest																		
	Treating chocking of child																		
	Treating child dyspnea																		

	Treating child seizure																		
	Treating child fever																		
4.22. Geriatric emergency	Treating elderly injury																		
	Treating elderly falling																		
	Treating elderly acute disease																		
	Treating elderly delirium																		
4.23. Environmental emergency	Treating heat emergency																		
	Treating cold emergency																		
	Treating bite & sting																		
	Treating of hazardous material exposure																		
4.24. Responding to special patient	Treating abused child																		
	Treating abused elderly																		
	Treating sexual assault patient																		
	Treating crime scene patient																		
	Responding to hostile behavior & self-harm																		
	Responding to dying & death																		

This is a survey for Patient Transportation. Please mark "○" for the questions that apply according to the criteria below.

Frequency					
①	②	③	④	⑤	⑥
0-10 %	11-30 %	31-50 %	51-70 %	71-90 %	90-100 %

Importance					
①	②	③	④	⑤	⑥
0-10 %	11-30 %	31-50 %	51-70 %	71-90 %	90-100 %

Difficulty					
①	②	③	④	⑤	⑥
0-10 %	11-30 %	31-50 %	51-70 %	71-90 %	90-100 %

Duty Ⅴ																			
Patient Transportation																			
Task	Task Elements	Frequency						Importance						Difficulty					
		①	②	③	④	⑤	⑥	①	②	③	④	⑤	⑥	①	②	③	④	⑤	⑥
5.1 Transporting	Applying body mechanics																		
	Applying c-collar																		
	Applying stretcher																		
	Applying KED																		
	Applying emergency urgent non-urgent move																		
	Transporting patient to treatable medical institution																		
5.2. Communicating	Applying principles of radio communication																		
	Providing information of patient																		
	Requesting direct medical control																		
5.3.Documenting	Documenting pre-hospital care report																		
	Documenting cardiac arrest care form																		
	Documenting cardiovascular care form																		
	Documenting major trauma care form																		
	Documenting infectious disease &																		

	hazmat report																		
	Documenting refusal of emergency care form																		
	Documenting advice and consent form																		
	Documenting hospital medical report																		
5.4. Transferring	Face-face reporting																		
	Reporting on special patient																		

This is a survey for Medical Assisting. Please mark "○" for the questions that apply according to the criteria below.

Frequency					
①	②	③	④	⑤	⑥
0-10 %	11-30 %	31-50 %	51-70 %	71-90 %	90-100 %

Importance					
①	②	③	④	⑤	⑥
0-10 %	11-30 %	31-50 %	51-70 %	71-90 %	90-100 %

Difficulty					
①	②	③	④	⑤	⑥
0-10 %	11-30 %	31-50 %	51-70 %	71-90 %	90-100 %

Duty Ⅵ																			
Medical Assisting																			
Task	Task Elements	Frequency						Importance						Difficulty					
		①	②	③	④	⑤	⑥	①	②	③	④	⑤	⑥	①	②	③	④	⑤	⑥
6.1. Applying KTAS	Triaging adult patient(KTAS																		
	Triaging child patient(KTAS																		
	Triaging trauma patient(KTAS																		
	Triaging non-trauma patient(KTAS																		
	Triaging special patient(KTAS																		
6.2. Monitoring	Checking BP for inpatient																		
	Checking pulse for inpatient																		
	Checking respiration for inpatient																		
	Checking body temperature for inpatient																		
	Checking SPO2·EtCO2 for inpatient																		
	Checking ECG for inpatient																		
	Drawing venous blood for inpatient																		
	Drawing arterial blood for inpatient																		
6.3. Lifting & moving	Moving patient in hospital																		
	Moving patient from ambulance to hospital																		

6.4. Inpatient treatment	Applying ET-tube																		
	Assisting foley catheterization																		
	Assisting L-tube insertion																		
	Assisting chest tube insertion																		
	Assisting thoracic depression																		
	Assisting tracheostomy																		
	Assisting paracentesis																		
	Assisting enema																		
	Securing a vein line																		
	Assisting central venous catheterization																		
	Assisting IM & SQ																		
	Assisting IO																		
	Applying CPR																		
	Assisting manual defibrillation																		
	Assisting synchronized cardioversion																		
	Assisting of intravenous administration Epinephrine																		
	Assisting of intravenous administration Atropine																		
	Assisting of intravenous administration Lidocaine																		
	Assisting of intravenous administration Amiodarone																		
	Administratingaspirin																		
	Assisting administration of non-narcotic analgesic																		
	Assisting ligation & cutting umbilical cord																		
	Assisting reduction & splinting																		

	fracture, dislocation																		
	Assisting operation																		
	Opening & maintaining airway																		
	Administrating O2																		
	Assisting hyperbaric O2 therapy																		
	Assisting wound treatment																		
	Assisting burn treatment																		
	Applying heat & cold																		

This is a survey for Disaster Medicine Management. Please mark "○" for the questions that apply according to the criteria below.

Frequency					
①	②	③	④	⑤	⑥
0-10 %	11-30 %	31-50 %	51-70 %	71-90 %	90-100 %

Importance					
①	②	③	④	⑤	⑥
0-10 %	11-30 %	31-50 %	51-70 %	71-90 %	90-100 %

Difficulty					
①	②	③	④	⑤	⑥
0-10 %	11-30 %	31-50 %	51-70 %	71-90 %	90-100 %

Duty Ⅶ																			
Disaster Medicine Management																			
Task	Task Elements	Frequency						Importance						Difficulty					
		①	②	③	④	⑤	⑥	①	②	③	④	⑤	⑥	①	②	③	④	⑤	⑥
7.1. Monitoring disaster	Determining level of disaster																		
	Determining type of disaster																		
	Determining nature of disaster																		
7.2. Managing disaster	Applying principles of disaster management																		
	Spreading information of disaster																		
	Managing hospital disaster																		
	Managing disaster supplies																		
7.3. Triaging MCI	Triaging adult victim																		
	Triaging child victim																		
	Triaging contaminated & infected victim																		
	Documenting triage tag																		
7.4. Performing in disaster department	Performing task of first-arrival team																		
	Performing task of disaster center																		
	Performing task of disaster headquarters																		
	Performing task of incident command unit																		

	Performing task of treatment unit																		
	Performing task of transportation unit																		
	Performing task of prompt-response team																		
	Performing task of DMAT																		
	Performing task of emergency medical support center																		
7.5. Responding to disaster	Responding to storm & flood damage																		
	Responding to large fire																		
	Responding to chemical disaster																		
	Responding to radioactive accident																		
	Responding to pandemic																		
	Responding to traffic accidents with MCI																		
7.6. Disaster psychological support	Screening for psychological support																		
	Helping for psychological support																		

This is a survey for Operations. Please mark "○" for the questions that apply according to the criteria below.

Frequency					
①	②	③	④	⑤	⑥
0-10 %	11-30 %	31-50 %	51-70 %	71-90 %	90-100 %

Importance					
①	②	③	④	⑤	⑥
0-10 %	11-30 %	31-50 %	51-70 %	71-90 %	90-100 %

Difficulty					
①	②	③	④	⑤	⑥
0-10 %	11-30 %	31-50 %	51-70 %	71-90 %	90-100 %

Duty Ⅷ																			
Operations																			
Task	Task Elements	Frequency						Importance						Difficulty					
		①	②	③	④	⑤	⑥	①	②	③	④	⑤	⑥	①	②	③	④	⑤	⑥
8.1.Managing situation	Providing information on medical institutions																		
	Responding to refusal of care & transfer																		
	Refusing emergency care & transportation																		
	Discontinuing & terminating resuscitation																		
8.2.Managing resource	Applying QM																		
	Organizing work schedule																		
	Driving ambulance																		
	Maintaining ambulance																		
	Maintaining equipment & supplies																		
	Knowing resource & EMS of area																		
8.3. Controlling infection	Applying cleaning, disinfection, & sterilization																		
	Disinfecting ambulance																		
	Controlling infection of EMTs & paramedics																		

	Responding to infectious disease																		
	Applying BSI																		

This is a survey for Self-Improvement. Please mark "○" for the questions that apply according to the criteria below.

Frequency					
①	②	③	④	⑤	⑥
0-10 %	11-30 %	31-50 %	51-70 %	71-90 %	90-100 %

Importance					
①	②	③	④	⑤	⑥
0-10 %	11-30 %	31-50 %	51-70 %	71-90 %	90-100 %

Difficulty					
①	②	③	④	⑤	⑥
0-10 %	11-30 %	31-50 %	51-70 %	71-90 %	90-100 %

Duty Ⅸ																			
Self-Improvement																			
Task	Task Elements	Frequency						Importance						Difficulty					
		①	②	③	④	⑤	⑥	①	②	③	④	⑤	⑥	①	②	③	④	⑤	⑥
9.1. Training	Training emergency care																		
	Training CPR																		
	Training clinical & squad trainee																		
9.2. Self-care	Maintaining health																		
	Managing stress																		
9.3. Maintaining professionalism	Participating in case-review																		
	Participating in continuing education																		
	Obtaining relevant certificates																		
	Joining organizations of professionals																		
	Participating in research activities																		
	Following regulations																		
	Following code of ethics																		

Thank you very much for answering the survey.

please check the next page.
